# Anti‐malarial drugs: Mechanisms underlying their proarrhythmic effects

**DOI:** 10.1111/bph.15959

**Published:** 2022-10-20

**Authors:** Khalil Saadeh, Nakulan Nantha Kumar, Ibrahim Talal Fazmin, Charlotte E. Edling, Kamalan Jeevaratnam

**Affiliations:** ^1^ Faculty of Health and Medical Sciences University of Surrey Guildford UK; ^2^ School of Clinical Medicine, Addenbrooke's Hospital University of Cambridge Cambridge UK; ^3^ Bristol Medical School University of Bristol Bristol UK

**Keywords:** anti‐l drugs, malaria, arrhythmia, artemisinin‐based combination therapy, cardiotoxicity, chloroquine, ion channels, malaria

## Abstract

Malaria remains the leading cause of parasitic death in the world. Artemisinin resistance is an emerging threat indicating an imminent need for novel combination therapy. Given the key role of mass drug administration, it is pivotal that the safety of anti‐malarial drugs is investigated thoroughly prior to widespread use. Cardiotoxicity, most prominently arrhythmic risk, has been a concern for anti‐malarial drugs. We clarify the likely underlying mechanisms by which anti‐malarial drugs predispose to arrhythmias. These relate to disruption of (1) action potential upstroke due to effects on the sodium currents, (2) action potential repolarisation due to effects on the potassium currents, (3) cellular calcium homeostasis, (4) mitochondrial function and reactive oxygen species production and (5) cardiac fibrosis. Together, these alterations promote arrhythmic triggers and substrates. Understanding these mechanisms is essential to assess the safety of these drugs, stratify patients based on arrhythmic risk and guide future anti‐malarial drug development.

**Abbreviations**
I_Ca‐L_
: inward depolarizing Ca^2+^ current
I_K1_
: inwardly rectifying background K^+^ current
I_KATP_
: inwardly rectifying ATP‐sensitive K^+^ current
I_Kr_
: rapidly activating component of the delayed K^+^ rectifier current
I_Ks_
: slowly activating component of the delayed K^+^ rectifier current
I_Na_
: fast Na^+^ current
I_Na‐L_
: late sodium current
I_to_
: transient outward K^+^ current

## 1 INTRODUCTION

Malaria is a vector‐borne parasitic tropical disease caused by protozoan *Plasmodium spp* transmitted via infected female *Anopheles* mosquitoes to humans through their bites (WHO, 2016). Though malaria‐related morbidity and mortality have been reported to have declined worldwide, there has been recent evidence to suggest that the decline in malaria burden, particularly the rate of reduction in mortality, has reduced and may have even stagnated from 2016 to 2018 compared with earlier this decade (Feachem et al., 2019; WHO, 2019). In the year 2017 alone, a total of 219 million cases and 435,000 deaths secondary to malaria were recorded (Feachem et al., 2019). We are still far from elimination despite a vast array of robust measures implemented by the global malaria community such as increased financial investment and development of new treatment regimens (Feachem et al., 2019; WHO, 2016).

As such, malaria remains the leading parasitic cause of death globally and its burden is felt severely in endemic areas, usually regions in low middle income countries (LMICs) such as sub‐Saharan Africa, where it has been reported that >1200 children lose their lives to the disease daily (Feachem et al., 2019). Table 1 outlines endemic regions and treatment regimens used based on the WHO Global Malaria Programme status report. Currently, an estimated 3.3 billion people across >90 countries and 125 million travellers are at risk of the disease and there are still circa 200 million cases per year, with number of deaths in the hundreds of thousands globally (Buck & Finnigan, 2020).

**TABLE 1:** Endemic regions and respective artemisinin‐based combination therapy (ACT) regimens used based on the WHO Global Malaria Programme status report (Artemisinin resistance and artemisinin‐based combination therapy efficacy, WHO STATUS REPORT of the Global Malaria Programme, 2019)

Region	Countries	*Plasmodium sp* strain targeted	Treatment regimen
Africa	Most African countries	*P. falciparum*	artemether‐lumefantrine (AL) (1st Line)
			artesunate‐amodiaquine (AS‐AQ) (1st Line)
			dihydroartemisinin‐ piperaquine (DHA‐PPQ)
	Some African countries Ethiopia, Madagascar and Mauritania	*P. vivax*	chloroquine (CQ)
			artemether‐lumefantrine (AL)
America	Countries in the Amazon region	*P. falciparum*	artemether‐lumefantrine (AL) (1st Line)
			artesunate‐ mefloquine (AS‐MQ) (1st Line)
	Guatemala, Haiti, Honduras and Nicaragua	*P. vivax*	chloroquine (CQ) (1st Line)
South‐East Asia	Bhutan, Nepal and Timor‐Leste	*P. falciparum*	artemether‐lumefantrine (AL) (1st Line)
	Indonesia	*P. falciparum*	dihydroartemisinin‐ piperaquine (DHA‐PPQ)
	Bangladesh	*P. falciparum*	artemether‐lumefantrine (AL) (1st Line)
			artesunate‐amodiaquine (AS‐AQ) (1st Line)
			artesunate‐ mefloquine (AS‐MQ) (1st Line)
			dihydroartemisinin‐ piperaquine (DHA‐PPQ) (1st Line)
	India		artemether‐lumefantrine (AL) (1st Line)
			artesunate + sulfadoxine‐pyrimethamine (AS+SP) 1st Line)
	Thailand		dihydroartemisinin‐ piperaquine (DHA‐PPQ) (1st Line)
			artesunate‐pyronaridine (AS‐PY) (1st Line)
	Myanmar		artemether‐lumefantrine (AL) (1st Line)
			artesunate‐ mefloquine (AS‐MQ) (1st Line)
			dihydroartemisinin‐ piperaquine (DHA‐PPQ) (1st Line)
	Bangladesh, Bhutan, the Democratic People's Republic of Korea, India, Myanmar, Nepal, Sri Lanka and Thailand	*P. vivax*	chloroquine (CQ) (1st Line)
	Indonesia	*P. vivax*	dihydroartemisinin‐ piperaquine (DHA‐PPQ) (1st Line)
	Timor‐Leste	*P. vivax*	artemether‐lumefantrine (AL) (1st Line)
Eastern Mediterranean region	Afghanistan, Djibouti, Pakistan, Somalia, Sudan	*P. falciparum*	artemether‐lumefantrine (AL) (1st Line)
	Somalia, Sudan	*P. vivax*	artemether‐lumefantrine (AL) (1st Line)
	Afghanistan, Djibouti, Iran (Islamic Republic of), Pakistan, Saudi Arabia, Yemen	*P. vivax*	chloroquine (CQ) (1st Line)
Western Pacific Region	Cambodia	*P. falciparum*	artesunate‐ mefloquine (AS‐MQ) (1st Line)
	Viet Nam	*P. falciparum*	dihydroartemisinin‐ piperaquine (DHA‐PPQ) (1st Line)
	Lao People's Democratic Republic, Malaysia, Papua New Guinea, Solomon Islands and Vanuatu	*P. vivax*	artemether‐lumefantrine (AL) (1st Line)
	China, the Republic of Korea and Viet Nam	*P. vivax*	chloroquine (CQ) (1st Line)
	Philippines	*P. vivax*	chloroquine (CQ) (1st Line)
			artemether‐lumefantrine (AL) (1st Line)
	Cambodia	*P. vivax*	artesunate‐ mefloquine (AS‐MQ) (1st Line)

## 2 ANTI‐MALARIAL DRUGS AND CARDIOTOXICITY

Cardiac complications in malaria could be caused by both the pathophysiological mechanisms of the infection itself or as a side effect of anti‐malarial treatment regimens. Although reports that cardiovascular complications of malaria and its treatment are relatively uncommon, it is worth noting that 90% of malaria deaths occur in African regions that are extremely scarce of resources and, hence, could indeed be subject to under‐diagnosis and under‐reporting (Gupta et al., 2021). A major concern regarding the wide‐scale use of combination therapy is the potential risk of adverse cardiac effects (Haeusler et al., 2018). There are several cardiac implications of quinoline and structurally similar anti‐malarial drugs documented such as bradycardia, hypotension, heart block and various ECG abnormalities. Anti‐malarial drugs have been associated with a wide range of adverse effects. Table 2 summarises reported side effects of commonly used anti‐malarial drugs.

**TABLE 2:** Side effects of anti‐malarial drugs as reported in the British National Formulary (BNF; Joint Formulary Committee, 2021)

Class of anti‐malarial	Antimalarial	Adverse drug effect
Quinolone derivative (4‐aminoquinolones)	Chloroquine	Cardiac: cardiomyopathy, atrioventricular block, hypotension, prolongation of QT interval
		Respiratory: interstitial lung disease
		Gastrointestinal disturbances: abdominal pain, nausea, vomiting
		Neurological: vision changes, movement disorders
		Haematological: agranulocytosis, thrombocytopenia, neutropenia, agranulocytosis
		Others: psychotic states, hallucination, anxiety, confusion, hearing impairment, insomnia
	Piperaquine **(**Artenimol with piperaquine phosphate)	Cardiac: arrhythmias, prolongation of QT interval, cardiac conduction disorder
		Gastrointestinal: loss of appetite, abdominal pain, hepatic disorders, nausea, vomiting
		Neurological: headache, dizziness, seizures
		Haematological: eosinophilia, leucocytosis, thrombocytopenia, thrombocytosis, anaemia
		Others: fever, lymphadenopathy, myalgia, arthralgia, cough, epistaxis
Quinolone derivative (4‐methanolquinolines)	Quinine	Cardiac: prolongation of QT interval, atrioventricular conduction disorders
		Respiratory: asthma, bronchospasm
		Gastrointestinal: abdominal pain, nausea, vomiting
		Neurological: cerebral impairment, coma, exacerbation of myasthenia gravis
		Haematological: agranulocytosis, haemoglobinuria, haemolytic uraemic syndrome, pancytopenia, thrombocytopenia
		Others: haemoglobinuria, renal impairment, agitation, vertigo, confusion
	Mefloquine	Cardiac: cardiac conduction abnormalities, chest pain, hypertension, hypotension, palpitations, syncope
		Respiratory: dyspnoea, pneumonia, pneumonitis
		Gastrointestinal: diarrhoea, nausea, vomiting, loss of appetite, pancreatitis
		Neurological: dizziness, headache, drowsiness, encephalopathy, memory loss, seizure
		Haematological: agranulocytosis, aplastic anaemia, thrombocytopenia
		Others: Renal impairment, arthralgia, anxiety, depression, sleep disturbances, confusion, mood disorders, tremor, vertigo
Quinolone derivative (8‐Aminoquinoline)	Primaquine	Cardiac: arrhythmias, dizziness, prolongation of QT interval
		Gastrointestinal: nausea and vomiting
		Haematological: haemolytic anaemia, leucopenia, methaemoglobinaemia
	Tafenoquine	Dizziness, nausea and vomiting
Antifolates	Atovaquone proguanil	Cardiac: dizziness, palpitations, tachycardia
		Gastrointestinal: loss of appetite, diarrhoea, nausea, vomiting, abdominal pain, hepatic disorders
		Neurological: headache
		Haematological: depression
		Others: hyponatraemia, vasculitis, Stevens‐Johnson syndrome
	Atovaquone with proguanil hydrochloride	Cardiac: prolonged QT interval, dizziness
		Respiratory:
		Gastrointestinal: loss of appetite, abdominal pain, diarrhoea
		Others: athralgia, cough, myalgia
Artemisinin combination therapy (ACT)	Artemether with lumefantrine	Cardiac: arrhythmias, prolonged QT interval, cardiac conduction abnormalities
		Gastrointestinal: loss of appetite, abdominal pain, vomiting, hepatic disorders
		Neurological: headache, seizure
		Haematological: anaemia, eosinophilia, leucopenia, leucocytosis, thrombocytosis, neutropenia
		Others: conjunctivitis, fever, arthralgia, myalgia, lymphadenopathy

However, the prime concern of the global malaria community is potential proarrhythmic effects, especially of prolonged QT‐interval, which represents a prolonged ventricular action potential duration and delayed repolarisation. It is associated with increased risk of potentially fatal polymorphic ventricular tachycardia torsades de pointes (TdP), ventricular fibrillation and sudden cardiac death (Haeusler et al., 2018).

However, although the QT interval is commonly accepted as a crucial marker for drug‐induced torsades de pointes risk, it is worth noting that the relationship between the QT interval and these arrhythmias remains unclear, with evidence suggesting that the QT interval is not a specific marker for torsades de pointes (Shah & Hondeghem, 2005; Traebert & Dumotier, 2005). Indeed, it has been suggested that the apparent exclusive association between prolonged QT interval and torsades de pointes is a *priori* the consequence of torsades de pointes definition (Shah & Hondeghem, 2005). Indeed, not all drugs that prolong the QT interval are associated with torsades de pointes. Torsades de pointes does not develop invariably in all individual with equivalent prolongation of QT interval, not all drugs that cause QT prolongation to equivalent levels are associated with the same risk of torsades de pointes and many cases of drug‐induced torsades de pointes have been reported in patients with a normal QT‐interval (Shah & Hondeghem, 2005). This highlights a fundamental over‐reliance on the QT‐interval to assess drug cardiac safety. Furthermore, methodological pitfalls and confounders in QT interval measurements further complicate this and include (i) co‐morbidities, such as cardiac disease and systemic physiological disturbances (Haeusler et al., 2018), (ii) biological variance based on gender, age and population based QTc normal ranges, (iii) heterogeneity in the measurement of the QT interval (van Vugt et al., 1999), denoted by time point of measurement (diurnal variation) and correction for heart rate (Fridericia's vs. Bazett's) and (iv) the potential pharmacokinetic interaction posed by co‐medication. Therefore, reliance on QT interval alone is not sufficient and further experimental and clinical investigations are necessary to assess arrhythmic risk of pharmacological agents especially anti‐malarial drugs.

## 3 THE PROARRHYTHMIC MECHANISMS OF ANTI‐MALARIAL DRUGS

Despite the common use of anti‐malarial drugs, their cardiotoxic effects remain poorly understood. Of those effects, the most widely reported is disruption to cardiac electrophysiology resulting in arrhythmias. Here, we explore the potential underlying arrhythmogenic mechanisms of anti‐malarial drugs particularly quinoline‐based anti‐malarial drugs, which represent some of the most commonly used and studied group (Haeusler et al., 2018). Moreover, certain anti‐malarial drugs such as chloroquine and hydroxychloroquine are now being investigated for and used in other conditions including rheumatoid arthritis and systemic lupus erythematosus, anti‐cancer therapy and pulmonary hypertension (Kamat & Kumari, 2021). Most recently, it has controversially been used in coronavirus disease 2019 (COVID‐19) (Kamat & Kumari, 2021). Nonetheless, cardiotoxic proarrhythmic effects have been a major concern in treating malaria and in all other repurposing of anti‐malarial drugs (Chatre et al., 2018; Traebert & Dumotier, 2005). Furthermore, it is likely that the proarrhythmic effects of different anti‐malarial drugs likely share similar underlying mechanisms associated with pathological disruption of (1) action potential upstroke due to effects on the sodium (Na^+^) currents, (2) action potential repolarisation due to effects on the potassium (K^+^) currents, (3) cellular calcium (Ca^2+^) homeostasis and (4) cardiac tissue fibrosis. Table 3 summarises the ion channel blocking effects of anti‐malarial drugs. These mechanisms are also likely exacerbated via synergistic effects during combinational therapies, which are increasingly being used to combat the emergence of drug resistance. Therefore, understanding the underlying proarrhythmic molecular mechanisms is essential to assess the safety of those drugs, stratify patients based on arrhythmic risk and guide future anti‐malarial drug development.

**TABLE 3:** Summarising the ion channel blocking effects of different anti‐malarial drugs with relevant experimental evidence

Channel	Current	Inhibited by	Experimental studies
Na_V_1.5	I_Na_	Quinidine	(Caspi et al., 2009; De La Coussaye et al., 1992; Ducroq et al., 2007; Hara et al., 1994; Miller et al., 1989)
		Chloroquine	(Baba et al., 2004; Jordaan et al., 2021; Sánchez‐Chapula et al., 2001)
		Primaquine	(Orta‐Salazar et al., 2002)
	I_Na‐L_	Quinidine	(Burke et al., 1986; Carmeliet & Saikawa, 1982)
		Chloroquine	(L. Wu et al., 2008; M. Wu et al., 2019)
K_ir_2.1	I_K1_	Quinidine	(Hirota et al., 2000; Noujaim et al., 2011)
		Chloroquine	(El Harchi et al., 2009; Lopez‐Izquierdo et al., 2009; Noujaim et al., 2010; Rodríguez‐Menchaca et al., 2008; Sánchez‐Chapula et al., 2001; Sanson et al., 2019; Zhang et al., 2020)
K_V_4	I_to_	Quinidine	(Hirota et al., 2000)
		Chloroquine	(M. Wagner et al., 2010)
		Primaquine	(M. Wagner et al., 2010)
		Mefloquine	(Perez‐Cortes et al., 2015)
K_V_11.1 (hERG)	I_Kr_	Quinidine	(Balser et al., 1991; Hirota et al., 2000)
		Chloroquine	(Borsini et al., 2012; Delaunois et al., 2021; Sánchez‐Chapula et al., 2001; Szendrey et al., 2021; Traebert et al., 2004; Whittaker et al., 2021)
		Hydroxychloroquine	(Delaunois et al., 2021; Szendrey et al., 2021; Whittaker et al., 2021)
		Halofantrine	(Borsini et al., 2012; Sánchez‐Chapula et al., 2004; Tie et al., 2000; Traebert et al., 2004)
		Primaquine	(Kim et al., 2010)
		Mefloquine	(Borsini et al., 2012; Kang et al., 2001; Traebert et al., 2004)
		Lumefantrine (and desbutyl‐lumefantrine	(Borsini et al., 2012; Traebert et al., 2004)
		Dihydroartemisinin and Piperaquine phosphate (together or individually)	(Borsini et al., 2012)
K_V_7.1	I_Ks_	Quinidine	(Balser et al., 1991; Kang et al., 2001; T. Yang et al., 2013)
		Mefloquine	(El Harchi et al., 2010; Kang et al., 2001)
K_ir_6.2	I_KATP_	Chloroquine	(Noujaim et al., 2010; Ponce‐Balbuena et al., 2012)
Ca_V_1.2	I_Ca‐L_	Quinidine	(Michel et al., 2002)
		Chloroquine	(Delaunois et al., 2021; Sánchez‐Chapula et al., 2001; Tona et al., 1990)
		Hydroxychloroquine	(Delaunois et al., 2021)
		Mefloquine	(Coker et al., 2000)

### 3.1 Sodium (Na^+^) currents

The cardiac sodium channel Na_V_1.5, encoded by the *SCN5A* gene, is responsible for ventricular activation via phase 0 depolarisation of the action potential (Huang, 2017). Disruption in the Na^+^ current (I_Na_) will have significant consequences on ordered action potential propagation through the myocardium. Cardiac conduction velocity is largely determined by the maximum rate of membrane depolarisation (dV/dt)_max_, which in turn is determined by the I_Na_ current conducted by the Na_V_1.5 channel (Huang, 2017). Thus, Na_V_1.5 inhibition will compromise ventricular activation and cardiac conduction velocity. Reduced conduction velocity forms the arrhythmic substrate associated with re‐entrant arrhythmias, whereby the action potential wave fails to completely extinguish and causes re‐excitation of previously excited, but now recovered, regions resulting in circus movement of activation. For example, Brugada syndrome arises from a Na_V_1.5 heterozygous knock out causing decreased excitability and slowed conduction velocity (Huang, 2017).

Multiple electrocardiographic reports have associated anti‐malarial drugs with bradycardia, atrioventricular (AV) or intraventricular conduction abnormalities (Haeusler et al., 2018). Specifically, they have been shown to delay ventricular depolarisation resulting in widening of the QRS complex (Chatre et al., 2018; White, 2007). In a phase 1 randomised study design, using the Comprehensive *In Vitro* Proarrhythmia Assay (CiPA), they found that chloroquine prolonged QRS in a concentration‐dependent fashion (Vicente et al., 2019). These reports support an inhibitory effect of anti‐malarial drugs on Na_V_1.5.

**FIGURE 1:**
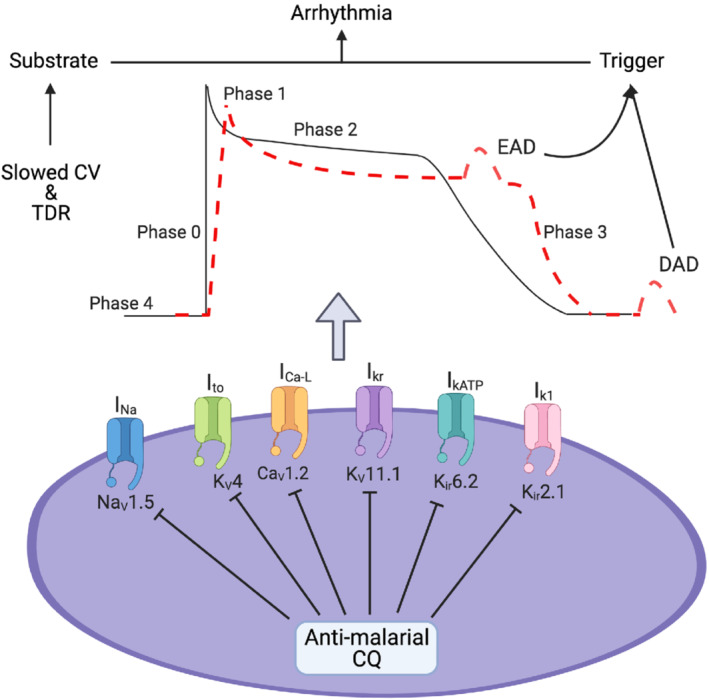
The reported effects of anti‐malarial drugs, especially chloroquine (CQ), on cell surface ionic currents. This includes inhibition of the fast Na^+^ current (I_Na_) conducted by Na_V_1.5 channels, the transient outward K^+^ current (I_to_) conducted by K_V_4 channels, the inward depolarizing Ca^2+^ current (I_Ca‐L_) conducted by Ca_V_1.2 channels, the rapidly activating component of the delayed rectifier current (I_Kr_) conducted by K_V_11.1 channels, the inwardly rectifying ATP‐sensitive current (I_KATP_) conducted by K_ir_6.2 channels and inwardly rectifying background K^+^ current (I_K1_) conducted by K_ir_2.1 channels. These alter the action potential waveform causing slowed membrane depolarisation and prolonged action potential duration. Together these changes in ionic currents promote the generation of early and delayed afterdepolarisations (EADs and DADs) arrhythmic triggers, as well as promoting the development of slowed conduction velocity (CV) and transmural dispersion of repolarisation (TDR) arrhythmic substrate resulting in increased risk of arrhythmia.

Experimental studies have also found that anti‐malarial drugs, particularly the quinoline‐based group, favour the inactivated state of the Na^+^ channel resulting in inhibition of I_Na_ (Mubagwa, 2020). In feline voltage‐clamped ventricular myocytes, chloroquine at clinically‐relevant concentrations of 0.3 to 10 μM decreased I_Na_ and reduced maximum upstroke velocity (Sánchez‐Chapula et al., 2001). In a canine model, the effect of protein kinase A to increase I_Na_ was suppressed by chloroquine application (Baba et al., 2004). This inhibitory effect was greater in cells from the epicardial zone of infarcted hearts compared to normal hearts, suggesting an exaggerated proarrhythmic effect of chloroquine in compromised hearts (Baba et al., 2004). In rat ventricles, primaquine produced a dose‐dependent block of I_Na_, increase in the time to peak current and depression of (dV/dt)_max_ during the upstroke of the action potential (Orta‐Salazar et al., 2002). Consistently, studies using a variety of experimental conditions demonstrated a frequency‐ and concentration‐dependent blockade of I_Na_, conduction delay and declining conduction velocity with quinidine. For example, in the presence of quinidine in guinea pig papillary muscle, a quiescent period followed by a train of stimulation produced an exponential decline in V_max_ and the decrease in V_max_ was enhanced by increasing stimulation frequency, that is, use‐dependent block (Hara et al., 1994). Hence, quinidine application to human embryonic stem cell derived cardiomyocytes, human embryonic kidney 293 (HEK‐293) cells and dog ventricles resulted in the development of arrhythmic substrates, whereby it decreased conduction velocity, prolonged the culture's total activation time and resulted in the generation of localised conduction block (Caspi et al., 2009; De La Coussaye et al., 1992; Ducroq et al., 2007). Similarly, in left atrial guinea pig hearts, quinidine at low rates of stimulation produced a transient positive inotropic effect and maximal action potential duration prolongation, whereas at high rates, it produced a negative inotropic effect and maximal (dV/dt)_max_ depression (Miller et al., 1989). This is explained by quinidine preferentially interacting with Na^+^ channels when they are in an open or inactivated state, but with K^+^ channels while they are closed (Miller et al., 1989). Thus, the majority of experimental reports indicate that quinidine exhibits Na_V_1.5 blocking effects associated with increased arrhythmic risk. However, some studies have shown that other anti‐malarial drugs do not have this effect. For example, automated whole‐cell patch‐clamp technique in HEK‐293 transfected with Na_V_1.5 cDNA measured Na_V_1.5 current and demonstrated that while chloroquine inhibited the Na_V_1.5 channel (with an IC_50_ of 8.48 μM), hydroxychloroquine did not (Jordaan et al., 2021). Consistent with this, in comparison with hydroxychloroquine, chloroquine caused higher incidence of proarrhythmic markers at therapeutic concentrations including aftercontraction, contraction failure, premature contraction and pause‐dependent arrhythmia in adult human primary cardiomyocytes (Jordaan et al., 2021). Additionally, in whole‐cell patch clamp studies in human myocytes obtained from human right atrial appendage, none of dihydroartemisinin (DHA; artenimol) alone, piperaquine phosphate (PQP) alone, dihydroartemisinin plus piperaquine phosphate or artemether plus lumefantrine blocked I_Na_, which was also demonstrated by the absence of QRS prolongation on ECG (Borsini et al., 2012). This highlights that different anti‐malarial drugs do not have the same effects on Na_V_1.5 and ventricular activation and hence will confer varying degrees of arrhythmic risk. As such, further experimental and clinical studies are required to clarify the effects of individual anti‐malarial drugs and anti‐malarial drug combinations on I_Na_ and therefore arrhythmic tendency. Figure 1 summarises the reported effects of anti‐malarial drugs, especially chloroquine, on cell surface ionic currents.

#### 3.1.1 Late sodium current

Interestingly, alongside the proarrhythmic effects of I_Na_ inhibition, anti‐malarial drugs may exert pro‐ or anti‐arrhythmic effects via Na_V_1.5 independent of I_Na_. Following the fast component of the Na^+^ current (I_Na_), the late sodium current (I_Na‐L_) contributes to action potential duration. In addition to prolonging the plateau phase of the cardiac action potential, I_Na‐L_ has been implicated in various mechanisms promoting arrhythmic triggers and substrate. Firstly, I_Na‐L_ has been shown to promote diastolic depolarisation triggering inappropriate action potentials in various pacemaker tissues including the sino‐atrial node, hence disrupting cardiac automaticity (Antzelevitch et al., 2014). Secondly, increased I_Na‐L_ prolongs membrane repolarisation and as such allows the development of early‐after depolarisations (EADs) through reactivation of voltage‐gated Ca^2+^ channels, which can trigger arrhythmic events (Huang, 2017; Saadeh, Shivkumar, & Jeevaratnam, 2020). The role of I_Na‐L_ in promoting early afterdepolarisations has been demonstrated in genetic conditions such as long QT syndrome type 3 (Huang, 2017; Saadeh, Achercouk, et al., 2020) and in acquired conditions such as heart failure (Horvath & Bers, 2014). Thirdly, increased I_Na‐L_ results in elevated intracellular Na^+^ concentrations that initially reduces the gradient for Ca^2+^ efflux through the sodium‐calcium exchanger. This results in a Ca^2+^ overload state, which predisposes to depolarising, transient inward currents triggering arrhythmias following full repolarisation known as delayed‐after depolarisations (DADs) (Antzelevitch et al., 2014). Fourthly, I_Na‐L_ has been implicated in promoting dispersion of repolarisation arrhythmic substrate for re‐entry arrhythmias. This is due to regional heterogeneity in I_Na‐L_ activity. Thus, M cells (microfold cells) and Purkinje fibres show greater I_Na‐L_ and longer action potential durations than endocardial or epicardial cells. Prolonged I_Na‐L_ in conditions such as heart failure result in increased dispersion of repolarisation (Maltsev et al., 2007). Consistently, experimental enhancement of I_Na‐L_ increases dispersion in repolarisation and increases torsades de pointes risk (Milberg et al., 2005).

Intriguingly, these proarrhythmic triggering effects of I_Na‐L_ appear to be exacerbated by a positive feedback loop with calcium/calmodulin‐dependent protein kinase II (CaMKII) whereby I_Na‐L_ increases CaMKII activity, which in turn increases I_Na‐L_ (Takla et al., 2020). For example, overexpression of CaMKII in rabbit and mouse ventricular myocytes enhanced intermediate inactivation, slowed recovery from inactivation and markedly increased intracellular Na^+^ concentration (Wagner et al., 2006). Similar to I_Na‐L_, CaMKII levels are increased in pathological conditions predisposing to arrhythmias such as ischaemia and heart failure (Kirchhefer et al., 1999) as well as in atrial fibrillation (Neef et al., 2010). In turn, activation of CaMKII has been associated with increased arrhythmic tendency via multiple mechanisms including phosphorylation of phospholamban and ryanodine receptor (RyR) leading to elevated Ca^2+^ levels and increasing Ca^2+^ leak during diastole and hence promoting spontaneous waves Ca^2+^ release and early afterdepolarisations (Curran et al., 2010). Inhibition of CaMKII abolished isoprenaline (isoproterenol) induced spontaneous Ca^2+^ waves and early afterdepolarisations in animal models of heart failure (Curran et al., 2010). CaMKII inhibition was also shown to reduce early afterdepolarisations in rabbit hearts (Anderson et al., 1998). Additionally, CaMKII activity promotes arrhythmic substrates. CaMKII reduces Na_V_1.5 availability inhibiting the peak I_Na_ (Takla et al., 2020; Wagner et al., 2006) as well as influencing the activity of multiple K^+^ currents (Mustroph et al., 2014). This causes reduced action potential conduction velocity, delayed repolarisation and prolonged effective refractory period predisposing to conduction block. Transmural dispersion of repolarization (TDR) also results from spatiotemporal heterogeneity in ion channel function, CaMKII activity, I_Na‐L_ magnitude and Na_V_1.5 availability. Together, this precipitates the substrate for re‐entry arrhythmia (Takla et al., 2020). Thus, in addition to the direct proarrhythmic effects of I_Na‐L_ on other currents, I_Na‐L_ can also influence ionic currents indirectly via CaMKII activity to increase arrhythmic tendency.

The effect of anti‐malarial drugs on I_Na‐L_ has infrequently been investigated. This is likely due to most studies demonstrating that anti‐malarial drugs prolong action potential duration rather than shorten consistent with inhibitory effects on K^+^ currents rather than I_Na‐L_ (see later). Nonetheless, one study demonstrated that quinidine, acting in a similar fashion to lidocaine, can shorten action potential duration in sheep Purkinje fibres via inhibition of an inward Na^+^ current (Carmeliet & Saikawa, 1982). In canine Purkinje fibres, quinidine also shortened action potential duration and effective refractory period, but this effect levelled off at midrange and was reversed at higher concentrations (Burke et al., 1986). Using whole cell voltage clamp recordings, one study examined the effect of chloroquine on I_Na‐L_ in the presence of either ATX‐II or veratridine used to alter I_Na‐L_ amplitude. Under both conditions, chloroquine reduced I_Na‐L_ whereby fractional current inhibition was 0.65 ± 0.04 and 0.64 ± 0.03 with ATX‐II and veratridine, respectively (M. Wu et al., 2019). The IC_50_ varied according to experimental conditions from 15.9 ± 1.2 μM (with ATX‐II, measured at the downward voltage ramp) to 43.5 ± 1.2 μM (with veratridine, measured at the downward voltage ramp) (Wu et al., 2019).

I_Na‐L_ inhibition by anti‐malarial drugs may counteract their other effects prolonging the action potential duration and QT interval. Hence exerting antiarrhythmic effects such as suppression of early afterdepolarisations and early afterdepolarisations arrhythmic triggers as well as reducing arrhythmic substrates. This may offer interesting insight into how anti‐malarial drugs influence arrhythmic tendency. One study has reported that proarrhythmic effects of quinidine including QT prolongation, increasing transmural dispersion of repolarisation and torsades de pointes induction were more prominent at low rather than high concentrations low concentrations of quinidine reduced I_Kr_, but higher concentrations reduced both I_Kr_ and I_Na‐L_ (L. Wu et al., 2008). Nevertheless, there is significant paucity of data regarding alteration of I_Na‐L_ by anti‐malarial drugs such that further research is necessary to confirm these observations in humans and whether these potentially antiarrhythmic effects occur at clinically relevant concentrations. It would also be highly interesting to investigate whether anti‐malarial drugs can influence the I_Na‐L_‐CaMKII pathway which, as discussed, has significant implications on arrhythmic tendency.

### 3.2 Potassium (K^+^) currents

Perhaps the most important electrocardiographic effect of anti‐malarial drugs is prolongation of the QT interval due to disruption of action potential repolarisation arising from inhibition of K^+^ currents. Prolonged QT interval predisposes to normally self‐terminating episodic polymorphic ventricular tachycardia, particularly torsades de pointes, where QRS complexes ‘twist’ around an isoelectric line in a sinusoidal fashion. Symptoms include palpitations, syncope and seizure‐like activity. Torsades de pointes has the potential to degenerate into ventricular fibrillation and/or sudden cardiac death (Kannankeril et al., 2010). In fact, the first drug to be clearly associated with QT prolongation, torsades de pointes and ventricular fibrillation (VF) was the anti‐malarial quinidine (Frey, 1918; Kannankeril et al., 2010).

Importantly, different anti‐malarial drugs produce varying degrees of QT prolongation. Chloroquine has repeatedly been shown to produce QT prolongation (Haeusler et al., 2018). Recently, in a cohort of 200 COVID‐19 patients, chloroquine and hydroxychloroquine treatment significantly prolonged the QT interval with seven patients (3.5%) requiring discontinuation of these medications due to corrected QT interval prolongation (Saleh et al., 2020). The only clinical reports of torsades de pointes and sudden cardiac death (SCD) associated with chloroquine use have been for its non‐malarial use where high doses are used for longer, such as in rheumatoid arthritis and systemic lupus erythematosus, or in cases of overdose (Haeusler et al., 2018). Causing far more potent QT prolongation is the anti‐malarial halofantrine, which is now considered to exert an unacceptable arrhythmogenic risk when used for malaria indications (Haeusler et al., 2018). As such, there have been multiple reports of extreme QT prolongation, torsades de pointes and sudden cardiac death associated with halofantrine (Bouchaud et al., 2009; Nosten et al., 1993). A number of artemisinin‐based combination therapies including dihydroartemisinin (DHA; artenimol)‐piperaquine, artesunate‐amodiaquine and artemether‐lumefantrine have been shown to significantly prolong the corrected QT interval (Funck‐Brentano et al., 2019). Contrastingly, artesunate‐pyronaridine administration studied in multiple studies has not been associated with significant QT prolongation (Funck‐Brentano et al., 2019; WANECAM, 2018). In addition to the specific anti‐malarial drug used, the length of exposure to the anti‐malarial is also important. For example, a dose‐dependent cumulative effect of chloroquine on the corrected QT interval was demonstrated with higher blood concentrations during the treatment period (Bustos et al., 1994). As such, low chloroquine doses were associated with significant QT prolongation when given over long periods of time suggesting a cumulative effect (Chatre et al., 2018).

#### 3.2.1 Inwardly rectifying current (I_K1_)

The normal membrane resting potential, represented as phase 4 of the action potential curve, is primarily maintained by an outward inwardly rectifying current (I_K1_) across inward rectifier K^+^ channels K_ir_2.1, K_ir_2.2 and K_ir_2.3. They also contribute to repolarisation and hence action potential duration (Huang, 2017). Their role in arrhythmogenesis is highlighted in Andersen's syndrome caused by an autosomal dominant loss‐of‐function mutation in the *KCNJ2* gene encoding the K_ir_2.1 (Plaster et al., 2001). This causes electrophysiological abnormalities characterised by prolonged action potential duration and QT interval and is associated with a significantly increased risk of cardiac arrhythmias including ventricular ectopics, polymorphic ventricular tachycardia, such as bidirectional ventricular tachycardia or torsades de pointes and ventricular fibrillation (Plaster et al., 2001). Thus, inhibition of I_K1_ will increase arrhythmic risk. This occurs by increasing incidence of arrhythmic triggers. Hence, I_K1_ block prolongs action potential duration permitting generation of early afterdepolarisations and decreases background polarising current, which normally offsets early afterdepolarisations (Sosunov et al., 2004).

When examining the effects of chloroquine on a variety of ionic currents in feline ventricular myocytes, chloroquine was most potent at blocking the I_K1_ current whereby 3 μM decreased I_K1_ by 67% at −100 mV (Sánchez‐Chapula et al., 2001). This suggests that I_K1_ is a major proarrhythmic target of anti‐malarial drugs. Using whole‐cell recordings of Chinese hamster ovary cells stably expressing human K_ir_2.1 channels, chloroquine significantly decreased I_K1_ current with an IC_50_ of 1.9 (1.8–2.1) μM (Sanson et al., 2019). Chloroquine dose‐ and voltage‐dependent inhibition of I_K1_ has been replicated in a variety of experimental conditions including mathematical modelling and a multitude of animal hearts such as guinea pig, mice, rabbits and sheep (El Harchi et al., 2009; Lopez‐Izquierdo et al., 2009; Noujaim et al., 2010; Sanson et al., 2019). Structural studies have found that chloroquine binds to and blocks the cytoplasmic pore domain, which is stabilised by negatively charged and aromatic amino acids within a central pocket (Rodríguez‐Menchaca et al., 2008; Zhang et al., 2020). It also interacts with negatively charged amino acids at the centre of the ion permeation vestibule of K_ir_2.1 (Noujaim et al., 2011; Rodríguez‐Menchaca et al., 2008). This makes chloroquine a more effective I_K1_ blocker than other anti‐malarial drugs such as quinidine, which also inhibits I_K1_ (Hirota et al., 2000; Noujaim et al., 2010; Noujaim et al., 2011). In contrast to these mechanisms causing acute reduction in I_K1_, one study has reported a role of chronic chloroquine application as a lysosomal inhibitor blocking the lysosomal degradation pathway in K_ir_2.1 breakdown hence increasing K_ir_2.1 expression (Jansen et al., 2008). The electrophysiological consequence of this mechanism is yet to be investigated, but it likely plays a role in limiting excessive alteration to the resting membrane potential and action potential duration (Mubagwa, 2020).

It is worth noting that although the I_K1_ blocking effects are proarrhythmic under normal conditions, the action potential lengthening effects of chloroquine may be antiarrhythmic under pathological conditions driven by significantly shortened action potential durations. This is evident in the case of short QT syndrome characterised by rapid cardiac repolarisation and potentially fatal arrhythmias. *In silico* mathematical modelling of human myocytes predicted that therapeutic concentrations of chloroquine would block abnormal K_ir_2.1 channel and normalising I_K1_, action potential duration and effective refractory period (Lopez‐Izquierdo et al., 2009), which was later confirmed using K_ir_2.1 transfected Chinese hamster ovary cells (El Harchi et al., 2009). Furthermore, chloroquine was also demonstrated to restore sinus rhythm in models of atrial fibrillation (Noujaim et al., 2011).

#### 3.2.2 Transient outward potassium current (I_to_)

Following stage 0 depolarisation, phase 1 repolarisation is conducted by K_V_4 channels, giving rise to the transient outward potassium current (I_to_). Inhibition of those channels reduces I_to_ and allows the plateau phase to occur at higher voltages. This permits an increase in action potential duration and I_Ca‐L_ and is therefore an important contributor to early afterdepolarisations generation triggering cardiac arrhythmias (Huang, 2017; Saadeh, Shivkumar, & Jeevaratnam, 2020).

Effects of anti‐malarial drugs on I_to_ predominantly show inhibition consistent with their proarrhythmic action potential duration prolonging effect and early afterdepolarisation generation (Perez‐Cortes et al., 2015; Wagner et al., 2010; Zhang et al., 2020). Nevertheless, in rat ventricular myocytes, the anti‐malarial drugs quinidine, chloroquine, primaquine and mefloquine all caused I_to_ inhibition (Hirota et al., 2000; Perez‐Cortes et al., 2015; Wagner et al., 2010). Specifically, chloroquine and primaquine were shown to reduce I_to_ amplitude via open‐channel block, accelerate its inactivation time constant and prolong recovery from inactivation (Wagner et al., 2010). This, however, occurred at high concentrations. For example, chloroquine blocked I_to_ amplitude (defined as the current inactivating during a test pulse of 600 ms duration) with an IC_50_ of 4.6 ± 0.9 mM, whereas the IC_50_ for I_to_ charge was 439 ± 63 μM and kinetic analysis for onset of block revealed K_d_ value of 520 ± 60 μM (Wagner et al., 2010). The effects of mefloquine were demonstrated on Chinese hamster ovary cells co‐transfected with human K_V_4.3 and its accessory subunit hKChIP2C by whole‐cell voltage‐clamp where mefloquine caused inhibition of I_to_ amplitude and modified steady‐state inactivation and recovery from inactivation (Perez‐Cortes et al., 2015). However, these findings have not been consistent across all studies. One study using voltage‐clamp techniques to measure I_to_ in feline ventricular myocytes found that chloroquine did not significantly affect the peak current amplitude or time course of I_to_ (Sánchez‐Chapula et al., 2001). Potential reasons for this discrepancy may involve use of experimental conditions including different drug concentrations as well as the use of different animal species, which will have different channel structures and hence different channel‐chloroquine interactions. This challenges alterations in I_to_ current as an important target in the proarrhythmic effects of anti‐malarial drugs.

#### 3.2.3 Delayed rectifier currents (I_Kr_ and I_Ks_)

Phase 3 repolarisation then drives the membrane potential towards the resting potential, thus determining action potential duration. Phase 3 repolarisation is in turn determined by voltage‐gated outward K^+^ currents predominantly involving the rapidly activating component of the delayed rectifier current (I_Kr_) conducted by K_V_11.1 (hERG) and the slow activating component of the delayed rectifier current (I_Ks_) conducted by the K_V_7.1 channel (Chiamvimonvat et al., 2017; Huang, 2017). Inhibition of those currents will delay repolarisation and hence prolong action potential duration promoting the generation of early afterdepolarisations. This also reduces the polarising current, which normally offsets delayed afterdepolarizations driven by the Na^+^‐Ca^2+^ exchanger (NCX). Additionally, due to regional differences in ion channel expression and function across the myocardium, disruption of repolarisation currents will result in transmural dispersion of repolarization owing to heterogeneities in recovery from excitability. This results in a unidirectional conduction block preventing the wave from self‐extinguishing (Chiamvimonvat et al., 2017). Thus, inhibition of repolarisation currents promotes early afterdepolarisations and early afterdepolarisations arrhythmic triggers and transmural dispersion of repolarisation arrhythmic substrate.

The most well‐known mechanism for drug‐induced action potential and QT prolongation is via blockade of the K_V_11.1 (hERG) channel carrying the I_Kr_ current. The arrhythmogenic potential of disruption to the I_Kr_ current is aptly demonstrated by loss‐of‐function mutations in the *HERG* gene resulting in Long QT Syndrome type 2 associated with polymorphic ventricular tachycardia, ventricular fibrillation and sudden cardiac death (Chiamvimonvat et al., 2017; Saadeh, Achercouk, et al., 2020). Hence, most drugs that induce torsades de pointes also shown to block I_Kr_ and result in QT prolongation; however, the opposite is not always true (Traebert & Dumotier, 2005). Although the relationship between I_Kr_ current inhibition and the likelihood of QT prolongation and torsades de pointes induction remains unclear, it is generally considered that a high potency of hERG channel and hence I_Kr_ current, inhibition is a risk factor for fatal arrhythmias particularly if the free therapeutic plasma concentration of the drug approximates its IC_50_ value for hERG inhibition (Traebert & Dumotier, 2005).

Using whole cell patch‐clamp technique on HEK‐293 cells transfected with the hERG channel found that halofantrine (IC_50_ 0.04 μM), chloroquine (IC_50_ 2.5 μM) and mefloquine (IC_50_ 2.6 μM), desbutyl‐lumefantrine (IC_50_ 5.5 μM) and lumefantrine (IC_50_ 8.1 μM) inhibited the channel in a concentration‐ and time‐dependent manner with halofantrine alone also blocking the hERG tail currents voltage‐dependently (Traebert et al., 2004). Of those drugs, halofantrine exhibited the greatest inhibitory and hence the greatest proarrhythmic effect (Traebert et al., 2004). Similarly, in Chinese hamster ovary cells stably expressing hERG channel, halofantrine blocked hERG tail currents elicited on repolarization to from +30 mV to −60 mV with an IC_50_ of 196.9 nM (the therapeutic plasma concentration range for halofantrine is 1.67–2.98 μM) (Tie et al., 2000). These results reflect clinical findings showing halofantrine is associated with significant dose‐dependent lengthening of QT interval (Abernethy et al., 2001). Further experiments in HEK‐293 cells stably expressing hERG channels showed that halofantrine, chloroquine, mefloquine, lumefantrine, piperaquine phosphate and dihydroartemisinin blocked the hERG current in a concentration‐dependent manner (Borsini et al., 2012). The IC_50_ for all of these drugs ranged from 3‐ to 30‐fold their C_max_, with halofantrine being the only exception with and IC_50_ lower than its C_max_. Intriguingly, dihydroartemisinin also decreased hERG trafficking reducing surface expression of the channel, though this was achieved at 300‐fold its C_max_ (Borsini et al., 2012). Thus, in rabbit ventricular wedge preparations of the same study, chloroquine significantly prolonged QT interval and increased torsades de pointes risk score measured from arrhythmic phenomenon including spontaneous early afterdepolarisations, R‐on‐T ectopic beats and torsades de pointes. However, the remaining anti‐malarial drugs exerted different effects on QT interval and torsades de pointes risk highlighting the varying electrophysiological consequences of anti‐malarial drugs. Hence, dihydroartemisinin did not increase QT interval or torsades de pointes risk, lumefantrine increased QT interval but did not induce torsades de pointes and piperaquine phosphate increased QT interval but no arrhythmic events observed and torsades de pointes risk score only significantly increased at 3 μM (>100‐fold its C_max_) (Borsini et al., 2012). As such, results of anti‐malarial drugs with lower I_Kr_ blocking potencies have been less consistent regarding QT prolongation. For example, studies have reported no significant QT prolongation following mefloquine administration (Lightbown et al., 2001).

Nevertheless, I_Kr_ blocking effects have been reported in a number of other studies for anti‐malarial drugs such as halofantrine (Sánchez‐Chapula et al., 2004), mefloquine (Kang et al., 2001), quinidine (Balser et al., 1991; Hirota et al., 2000), primaquine (Kim et al., 2010), chloroquine (Delaunois et al., 2021; Sánchez‐Chapula et al., 2001) and hydroxychloroquine (Delaunois et al., 2021). These include recent studies, largely motivated by repurposing chloroquine and hydroxychloroquine for COVID‐19 treatment, utilising patch clamp techniques, human induced pluripotent stem cell‐derived cardiomyocytes and mathematical modelling, such as human *in silico* drug trials, which implements the population of cell model methodology modelling human ventricular electrophysiology which showed that both chloroquine and hydroxychloroquine inhibited I_Kr_ [IC_50_ 1 μM and 3–7 μM, respectively (Delaunois et al., 2021)], exacerbated torsades de pointes indicators and promoted early afterdepolarisation generation (Delaunois et al., 2021; Montnach et al., 2021; Szendrey et al., 2021; Whittaker et al., 2021; Wong et al., 2021). Clinical studies involving patients receiving chloroquine and hydroxychloroquine for COVID‐19 treatment have, therefore, expectedly shown prolongation of QT interval in those patients with some cases terminating treatment due to excessive prolongation and incidence of cardiac arrhythmias including torsades de pointes (Diaz‐Arocutipa et al., 2021; Eftekhar et al., 2021). Furthermore, one study using rabbit ventricles directly demonstrated a link between quinidine application, QT prolongation, enhancement of transmural dispersion of repolarisation arrhythmic substrate and incidence of torsades de pointes (Wu et al., 2008) consistent with other studies showing quinidine increases transmural dispersion of repolarisation (Antzelevitch et al., 1999). Another study demonstrated prolonged exposure to quinidine prolonged action potential duration and increased incidence of delayed afterdepolarisation arrhythmic triggers (Wit et al., 1990).

On the other hand, the slow activating component of the delayed rectifier potassium current I_Ks_, conducted by the K_V_7.1 channel, has less frequently been implicated in drug‐induced QT prolongation and as such much less research has investigated the effects of anti‐malarial drugs on I_Ks_. Nevertheless, I_Ks_ still plays an important role in shaping the cardiac action potential and hence arrhythmic tendency. I_Ks_ appears to play a key role in cardiac repolarisation reserve forming a negative feedback mechanism that limits excessive repolarisation lengthening (Varró & Baczkó, 2011). Thus, when action potential duration is prolonged by other means (e.g., by reductions in I_Kr_ or I_k1_), I_Ks_ activation is favoured and acts to prevent further action potential duration lengthening. Hence, pharmacological inhibition of I_Ks_ might have significant proarrhythmic consequences as this protective mechanism is eliminated (Roden & Yang, 2005; Varró & Baczkó, 2011). This is evident in loss‐of‐function mutations of the KCNQ1 gene resulting in long QT syndrome type 1 significantly increasing the risk of torsades de pointes and sudden cardiac death (Huang, 2017; Saadeh, Shivkumar, & Jeevaratnam, 2020).

In contrast to findings in I_Kr_, studies indicate that anti‐malarial drugs do not significantly inhibit I_Ks_. In Xenopus oocytes, application of high concentration halofantrine (10 μM) did not significantly affect I_Ks_ (Sánchez‐Chapula et al., 2004). In feline ventricular myocytes, chloroquine did not block the I_Ks_ current (Sánchez‐Chapula et al., 2001). In rabbit ventricular wedge preparations, application of a variety of anti‐malarial drugs (chloroquine, piperaquine phosphate, dihydroartemisinin, piperaquine phosphate and dihydroartemisinin combination, artemether and lumefantrine combination) did not significantly block the I_Ks_ current producing less than 20% inhibition even at 100‐fold their C_max_ (Borsini et al., 2012). Both chloroquine and hydroxychloroquine have also been reported not to exert significant inhibitory effects on I_Ks_ in Chinese hamster ovary cells (Delaunois et al., 2021). Interestingly, this is not consistent across all studies. Patch‐clamped Chinese hamster ovary cells showed that mefloquine, in a concentration‐dependent manner, inhibited I_Ks_ amplitude (IC_50_ ~1 μM) and slowed current activation (El Harchi et al., 2010; Kang et al., 2001). Quinidine was also shown to block the KCNQ1 channel (Balser et al., 1991; Kang et al., 2001; Yang et al., 2013), though much less potently than mefloquine with reported IC_50_ values of 44 μM (Kang et al., 2001) and 19.4 μM (T. Yang et al., 2013).

#### 3.2.4 ATP‐sensitive K^+^ current (I_KATP_)

ATP‐sensitive sarcolemmal K^+^ channels (sarcK_ATP_) carry an inwardly rectifying K^+^ current and are primarily formed by K_ir_6.2 and SUR2A channels. They generally account for relatively little current due to inhibition by intracellular ATP (Huang, 2017). However, they are activated by a reduced ATP/ADP ratio and play an important role in the electrophysiological response to metabolic stress, for example, ischaemia (Saadeh & Fazmin, 2021). The high density of sarcK_ATP_ channels creates a situation whereby only 1% of those channels need to be activated in order to significantly shorten action potential duration and effective refractory period (Huang, 2017). Furthermore, opening of a large number of those channels drives cardiomyocyte membrane potential towards the potassium's equilibrium potential such that cardiomyocyte become hyperpolarised and unexcitable (Saadeh & Fazmin, 2021). This causes a ‘current sink’, which slows or block action potential propagation (Akar & O'Rourke, 2011). These changes promote re‐entrant arrhythmias (Akar & O'Rourke, 2011; Huang, 2017). Hence, gain‐of‐function mutations in sarcK_ATP_ channels result in a number of arrhythmic phenotypes including ventricular fibrillation (Nakaya, 2014).

Chloroquine was shown to inhibit I_KATP_ in HEK293 cells sing inside‐out patch‐clamp recordings (Ponce‐Balbuena et al., 2012). Similar findings were reported in mice, rabbits and sheep hearts (Noujaim et al., 2010). This inhibition occurred via two mechanisms including a fast‐onset effect via direct channel pore block resulting from the drug entering the channel pore from the cytoplasmic side at depolarised membrane voltages and a slow‐onset, voltage‐independent effect allosteric effects on channel gating and interaction with phosphatidylinositol 4,5‐bisphosphate (Noujaim et al., 2010; Ponce‐Balbuena et al., 2012). Interestingly, because I_KATP_ activation is generally considered proarrhythmic, then these inhibitory effects of chloroquine may exert antiarrhythmic effects. Hence, in rabbit ventricles pinacidil (I_KATP_ opener) was used to induce ventricular fibrillation. However, addition of chloroquine slowed and in some cases abolished, ventricular fibrillation (Noujaim et al., 2010).

Nevertheless, I_KATP_ activation is protective against ischaemia/reperfusion injury whereby hyperpolarisation of the membrane prevents hypoxia/reoxygenation‐induced Ca^2+^ overload, myocyte hypercontracture and death (Baczkó et al., 2004). In experiments using arterially perfused guinea pig right ventricular walls, reperfusion of glibenclamide‐treated tissues (I_KATP_ inhibitor) elicited arrhythmias (extrasystoles and tachycardia) and the preparations failed to recover mechanical function. Whereas pinacidil‐treated tissues underwent complete recovery of electrical and mechanical activity (Cole et al., 1991). This suggests that the previously discussed beneficial electrophysiological effect of I_KATP_ block can be offset by impairment of cardioprotection following I_KATP_ inhibition. These conflicting findings indicate that further research is required to clarify the electrophysiological consequences of I_KATP_ inhibition.

Overall, clinical and experimental studies demonstrate that anti‐malarial drugs inhibit a variety of K^+^ currents which leads to delayed repolarisation hence prolonging the action potential duration and the QT interval of the electrocardiogram which is a sensitive but nonspecific risk marker for the development of torsades de pointes (Zhang et al., 2020). Hence, anti‐malarial drugs promote arrhythmogenesis by increasing early afterdepolarisations and early afterdepolarisations arrhythmic triggers and transmural dispersion of repolarisation arrhythmic substrate.

### 3.3 Calcium (Ca^+^) currents

In addition to the proarrhythmic alterations of Na^+^ and K^+^ currents, anti‐malarial drugs have been shown to influence cell surface Ca^+^ entry and intracellular Ca^+^ homeostasis, both of which have important implications on cardiac function and arrhythmogenicity.

#### 3.3.1 L‐type Ca^+^ current (I_Ca‐L_)

In response to membrane depolarisation, transverse tubular voltage‐gated Ca_V_1.2 L‐type Ca^2+^ channels open and initiate excitation–contraction coupling through the influx of extracellular Ca^2+^, triggering RyR‐mediated Ca^2+^‐induced Ca^2+^ release. The inward depolarizing Ca^2+^ current (I_Ca‐L_) is responsible for the plateau phase 2 of the action potential waveform (Huang, 2017). These channels play an important role in arrhythmic mechanisms. In conditions of prolonged action potential duration, L‐type Ca^2+^ channels recover from inactivation in a still depolarised membrane allowing I_Ca‐L_ to produce further depolarisation initiating a positive feedback mechanism resulting in early afterdepolarisations potentially triggering inappropriate action potentials and hence arrhythmias (Huang, 2017). This appears to be the primary mechanism by which L‐type Ca^2+^ channels contribute to arrhythmogenesis. Thus, experiments have demonstrated that inhibition of these channels abolishes early afterdepolarisation generation and is associated with reduced arrhythmic risk (Madhvani et al., 2015). They, therefore, are important in antiarrhythmic therapy being the target of class IV antiarrhythmic drugs. It has also been suggested that in some patients with inducible ventricular tachycardias, Ca^2+^ channels can support slow conduction, especially in depolarised tissue, which forms the substrate for re‐entry arrhythmias (Shorofsky & Balke, 2001).

In voltage‐clamped cat ventricular myocytes, chloroquine blocked I_Ca‐L_ in a concentration‐dependent manner with a 32 ± 11% at 10 μM measured +10 mV, without altering the I_Ca‐L_‐V relationship (Sánchez‐Chapula et al., 2001). Other studies have also shown inhibitory effects of chloroquine (Delaunois et al., 2021; Tona et al., 1990), hydroxychloroquine (Delaunois et al., 2021), mefloquine (Coker et al., 2000), quinine (Michel et al., 2002) and quinidine (Michel et al., 2002) on I_Ca‐L_ current. Interestingly, these findings would suggest that these anti‐malarial drugs would exert an antiarrhythmic effect by suppressing early afterdepolarisation generation due to I_Ca‐L_ inhibition. However, this is unlikely to be an important antiarrhythmic mechanism of anti‐malarial drugs as what is important is not only effects on individual currents but on different currents relative to each other. Thus, considering the potency by which anti‐malarial drugs alter different currents, their proarrhythmic I_K_ current inhibition exceeds potential antiarrhythmic I_Ca‐L_ current inhibition. For example, in one study directly comparing the inhibitory potency of chloroquine on feline ventricular myocytes on different currents, chloroquine was most potent at blocking I_K1_ and least potent at blocking I_Ca‐L_ (Sánchez‐Chapula et al., 2001). Nevertheless, these I_Ca‐L_ blocking effects have also been associated with slowed pacemaker activity partially explaining atrio‐ventricular conduction block observed with anti‐malarial drug use (Mubagwa, 2020; Sánchez‐Chapula et al., 2001).

Furthermore, while effects of anti‐malarial drugs on I_Ca‐L_ may not play an important role in arrhythmogenesis, they can still exert cardiotoxic effects by compromising cardiac contractile function due to the central role of the L‐type Ca^2+^ channels in excitation–contraction coupling. Consistent with this, chloroquine was shown to have negative inotropic effects whereby it depressed the developed tension in atrial and ventricular tissue, and caused haemodynamic compromise in anaesthetised rats and rabbits (Hughes, 2020; Riou et al., 1989). Similar findings were reported in Langendorff‐perfused guinea pig ventricles where chloroquine decreased developed tension and hence reduced ventricular contraction (Tona et al., 1990). This negative inotropic effect (also reported in quinine, quinacrine and mefloquine) was antagonised by increases in extracellular Ca^2+^ demonstrating that the underlying mechanism is related to I_Ca‐L_ inhibition (Coker et al., 2000; Ikhinmwin et al., 1981; Tona et al., 1990).

#### 3.3.2 Intracellular Ca^+^ homeostasis

Moreover, the anti‐malarial chloroquine can influence intracellular Ca^2+^ homeostasis by inhibiting lysosomal function and autophagy. Chloroquine accumulates in lysosomes by ion trapping causing lysosomal alkalinisation and disrupted autophagic flux by decreasing autophagosome‐lysosome fusion (Mauthe et al., 2018; Mubagwa, 2020). In a rat model of pressure overload hypertrophy, chloroquine treatment significantly decreased markers of macroautophagy and chaperone‐mediated autophagy (Chaanine et al., 2015).

**FIGURE 2:**
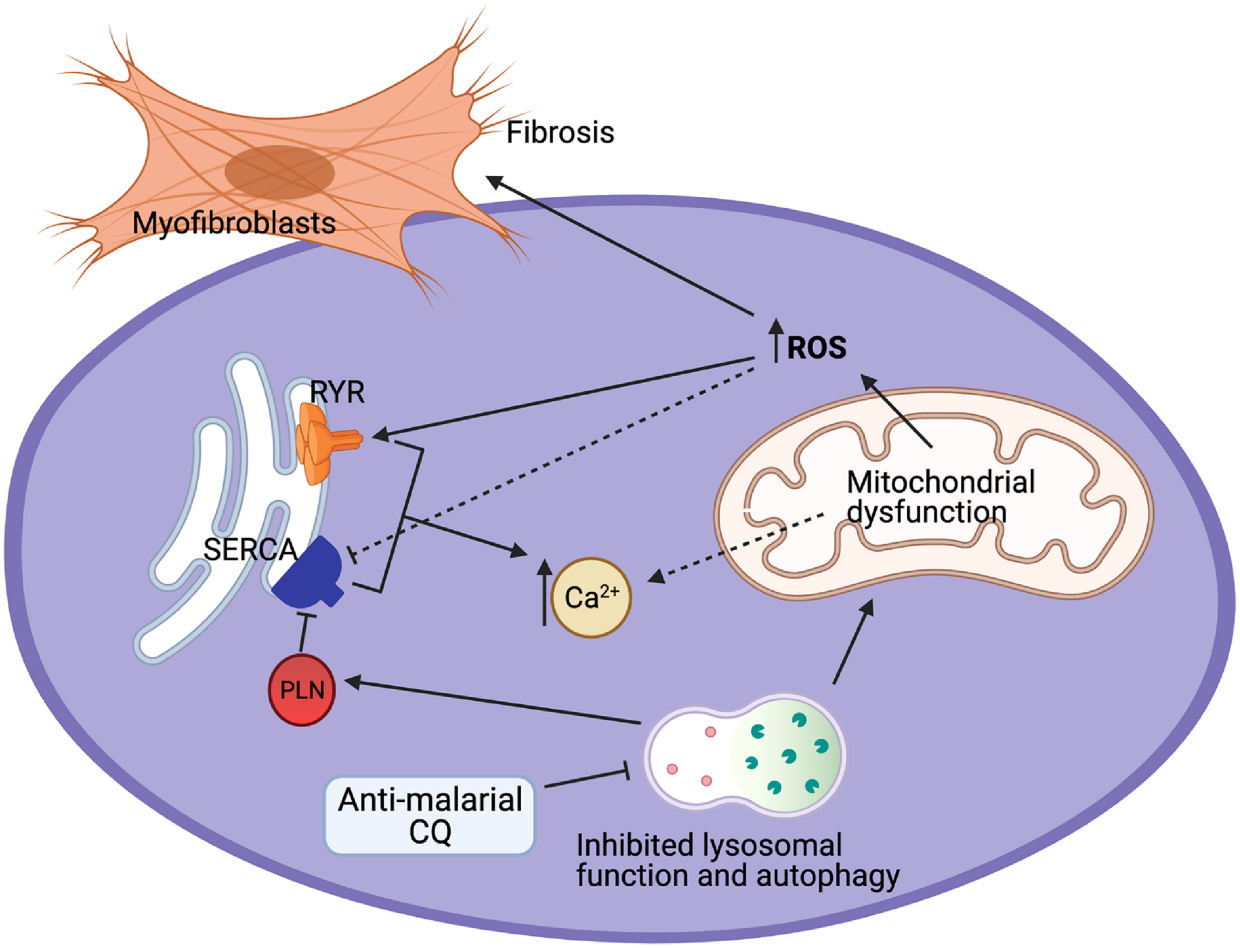
The reported intracellular effects of anti‐malarial drugs, especially chloroquine (CQ), on mitochondrial function and Ca^2+^ homeostasis. CQ accumulates in lysosomes and disrupts autophagosome‐lysosome fusion. This promotes phospholamban (PLN) accumulation which negatively regulates sarcoendoplasmic reticulum Ca^2+^‐ATPase (SERCA) function. It also results in significant disruption to mitochondrial structure and function with causing elevated intracellular Ca^2+^ and reactive oxygen species production (ROS). ROS in turn further increases intracellular Ca^2+^, for example, via oxidation of ryanodine receptor (RyR) and promotes development of cardiac fibrosis.

This influences intracellular Ca^2+^ by 1) directly altering expression levels of Ca^2+^ homeostasis proteins and 2) indirectly by disrupting mitochondrial function. In cultured mouse neonatal cardiomyocytes, chloroquine promoted endogenous phospholamban accumulation (Teng et al., 2015). Phospholamban binds to the sarcoendoplasmic reticulum Ca^2+^‐ATPase (SERCA) to negatively regulate its function. SERCA is critical to terminating the cardiac cycle through diastolic reuptake of cytosolic Ca^2+^ into the sarcoplasmic reticulum, which in turn influencing RyR2‐mediated systolic Ca^2+^ release (Huang, 2017; Saadeh, Achercouk, et al., 2020). Pathological alterations in SERCA function is associated with increased arrhythmic risk (Prunier et al., 2008; Saadeh, Achercouk, et al., 2020). With respect to mitochondrial function, chloroquine results in significant disruption to mitochondrial structure and function causing elevated intracellular Ca^2+^ and reactive oxygen species (ROS) production. Both of those cause proarrhythmic electrophysiological changes (Edling et al., 2019; Saadeh & Fazmin, 2021). Thus, in a rat model of pressure overload, chloroquine accentuated mitochondrial fragmentation and cristae destruction with a plethora of autophagosomes containing collapsed mitochondria and lysosomal lamellar bodies (Chaanine et al., 2015). Chloroquine also exaggerated pressure overload induced oxidative stress through the further decrease in the expression of manganese superoxide dismutase (Chaanine et al., 2015). Furthermore, chloroquine led to depolarisation of the mitochondrial membrane potential and increased production of mitochondrial ROS (Liu et al., 2019; Murphy et al., 2019). This was specifically shown to increase oxidation of the sarcoplasmic reticulum Ca^2+^ release channel RyR2, which in turn disturbed intracellular Ca^2+^ homeostasis and enhanced spontaneous Ca^2+^ release under β‐adrenoceptor stimulation resulting in increased arrhythmic tendency (Liu et al., 2019; Murphy et al., 2019). In addition to enhancing RyR2 activity, mitochondrial dysfunction and ROS also increase intracellular Ca^2+^ via SERCA inhibition (Saadeh & Fazmin, 2021; Xu et al., 1997) and through reduced mitochondrial Ca^2+^ storage capacity (Saadeh & Fazmin, 2021; Yang et al., 2015). However, these mechanisms are yet to be investigated in the context of chloroquine or other anti‐malarial drug use. Figure 2 summarises the reported intracellular effects of chloroquine on mitochondrial function and Ca^2+^ homeostasis.

The proarrhythmic consequences of elevated intracellular Ca^2+^ are clearly evident in arrhythmic syndromes arising from mutations in cellular Ca^2+^ handling component such as RyR2 as occurs in catecholaminergic polymorphic ventricular tachycardia (Saadeh, Achercouk, et al., 2020). Pathologically elevated intracellular Ca^2+^ increases arrhythmic risk by (1) promoting the activity of the electrogenic NCX resulting in the generation of delayed afterdepolarisation (Huang, 2017; Saadeh, Achercouk, et al., 2020), (2) Ca^2+^ cycling which has been associated with action potential duration alternans and spatiotemporal heterogeneities in repolarisation (Edwards & Blatter, 2014; Huang, 2017), (3) interacting with surface membrane Na_V_1.5 and connexin channels causing reduced conduction velocity (Huang, 2017; King et al., 2013) and (4) enhancing ROS production contributing to tissue fibrosis (Saadeh & Fazmin, 2021).

### 3.4 Cardiac tissue fibrosis

As discussed, chloroquine is associated with energetic dysfunction. In turn, mitochondrial dysfunction and accelerated ROS generation increases expression of pro‐fibrotic signalling molecules such as TGF‐β and its downstream effector connective tissue growth factor (CTGF) (Biernacka & Frangogiannis, 2011; Dai et al., 2009). These induce fibroblast proliferation, differentiation into myofibroblasts and the production of extracellular matrix components (Biernacka & Frangogiannis, 2011). Both clinical and experimental studies strongly associate fibrosis with atrial and ventricular arrhythmias (de Bakker et al., 1990; Hassink et al., 2003; Saadeh et al., 2021). For example, isolated Langendorff‐perfused human hearts with extensive infarction or dilated cardiomyopathy demonstrated increased vulnerability to triggering of ventricular tachycardia due to cardiac fibrosis facilitating re‐entry mechanisms (de Bakker et al., 1990; Wu et al., 1998). Fibrosis increases arrhythmic risk in multiple ways including slowed conduction velocity (Huang, 2017; King et al., 2013) and promotion of action potential duration alternans and dispersions of refractoriness causing unidirectional conduction block arrhythmic substrate (Spach et al., 1988; Turner et al., 2005).

It is likely that due to the chronic progressive nature of fibrotic change, fibrosis may not play an important part in the proarrhythmic mechanisms of anti‐malarial drugs in the acute setting. However, this becomes important when anti‐malarial drugs are repurposed for other conditions such as rheumatoid arthritis requiring longer term regimens of the drug (Dos Reis Neto et al., 2020). Additionally, these mechanisms are relevant during chloroquine administration in patients under high‐oxidative stress conditions, such as pathological myocardial hypertrophy or heart failure (Chaanine et al., 2015; Zhang et al., 2020).

Clinically, chronic chloroquine therapy can lead to cardiomyopathy characterised by hypertrophy and wall thickening and microscopic structural changes and associated with conduction disorders (Tönnesmann et al., 2013). As such, chloroquine at high doses exacerbated pressure overload hypertrophy and impaired cardiac contractility in rats (Chaanine et al., 2015; Zhang et al., 2020). Therefore, chronic chloroquine may exert additional proarrhythmic effects by promoting cardiac fibrosis.

## 4 COMBINATIONAL THERAPIES

### 4.1 Anti‐malarial drug resistance

Resistance to chloroquine and other older anti‐malarial drugs such as amino‐quinolines and antifolates has been a long‐standing issue which has prompted the use of combinational treatment to slow down the rising rate of resistance to anti‐malarial monotherapy (Conrad & Rosenthal, 2019; WHO, 2019). Thus, major progress has been made by the global malaria community to control malaria internationally via the use of highly efficacious artemisinin‐based combination treatments towards *P*. *falciparum* complemented by other control non‐pharmacological control measures (Feachem et al., 2019). In artemisinin‐based combination therapy, potent artemisinin, half‐life <1 h, is known to rapidly eradicate the bulk of the *Plasmodium* spp. The ‘partner drugs’ such as piperaquine, sulfadoxine‐pyrimethamine, lumefantrine, mefloquine and amodiaquine, which have a longer half‐life eradicate the remaining residual parasites over days to weeks (Haldar et al., 2018). It was promulgated that parasites that become resistant to artemisinins *de novo*, will be eliminated by longer half‐life partner drugs (Haldar et al., 2018). However, in 2008, there were concerning reports of resistance to artemisinin‐based combination therapy defined as treatment failures in the Greater Mekong Subregion of Southeast Asia (WHO, 2019). In contrast to the previous theory, reports have suggested that the duration for parasite clearance increases with artemisinin‐based combination therapy and this ‘delayed clearance phenotype’ could lead to both resistance against artemisinin as well as the partner drugs leading to multi‐drug resistance. Two mechanisms of drug resistance conferred by the parasite against artemisinin anti‐malarials have been suggested (1) the activation of the unfolded protein response and (2) proteostatic dysregulation of phosphatidylinositol 3‐kinase (PfPI3K) (Mbengue et al., 2015). Given the dynamic nature of drug resistance and the virulence of the parasite, there is an emerging concern that resistance to artemisinin‐based combination therapy and partner drugs may not only spread locally but internationally to endemic areas, such as South Africa where artemisinin‐based combination therapy are the current standard treatment (Conrad & Rosenthal, 2019; Haldar et al., 2018). Such a catastrophic eventuality not only highlights the urgency of resistance surveillance in this continent but encourages the investigation of more efficacious anti‐malarial drug combinations. However, in order to do this, an understanding of the potential adverse consequences, particularly arrhythmic risk, of those combinations needs to be investigated. Additionally, there is a mounting interest towards the development and wide use of triple artemisinin‐based combination therapy (van der Pluijm et al., 2020).

### 4.2 Anti‐malarial drug combinations

WHO outlines the first‐line treatments of malaria in its Global Malaria Program (GMP) status report based on each region (WHO, 2019). First line artemisinin‐based combination treatments currently used are dihydroartemisinin‐piperaquine, artemether‐lumefantrine, artesunate‐mefloquine, artesunate‐amodiaquine and artesunate plus sulfadoxine‐pyrimethamine (Table 1). The choice of anti‐malarial regimen used at a particular region is informed by therapeutic efficacy studies and data integrated drug efficacy surveillance, along with the presence of molecular markers of resistance, that is, *PFKELCH13* mutations (Mbengue et al., 2015; WHO, 2019).

Most research into the electrophysiological effects of anti‐malarial drug combinations is clinical and primarily focuses on alterations in the ECG QT interval. It has been demonstrated that combined therapy with mefloquine and halofantrine produces a prolongation in the QT interval that is greater than the prolongation from either drug alone, such that for any given plasma concentration of halofantrine, the QT interval is prolonged by addition of mefloquine (Lightbown et al., 2001; Nosten et al., 1993). However, other anti‐malarial drug combinations did not show exacerbated QT lengthening. In a study involving 200 patients who received artemether‐lumefantrine combination therapy no significant QT interval prolongation (Badshah et al., 2017). The relative safety of artemether‐lumefantrine therapy has been consistent across multiple clinical reports (Mhamilawa et al., 2020). Data from 15 trials showed that the frequency of QT interval prolongation in patients receiving artemether‐lumefantrine therapy ranged from 0.8% to 8.3% and was similar to or lower than that observed with chloroquine, mefloquine, or artesunate plus mefloquine; these changes were considerably less frequent than with quinine or halofantrine. Importantly, all patients with QT prolongation remained asymptomatic with no adverse clinical cardiac events were reported (Bakshi et al., 2000).

A randomised non‐inferiority trial treating uncomplicated malaria in African children compared dihydroartemisinin plus piperaquine phosphate to artemether‐lumefantrine treatment. It found that the proportion of patients with prolonged QTc interval at day 2 corrected by the Bazett's method was higher in the dihydroartemisinin plus piperaquine phosphate (9.1%) than the artemether‐lumefantrine group (6.9%) (Bassat et al., 2009) consistent with other studies (Saito et al., 2021; WANECAM, 2018). However, this was not confirmed when applying the Fridericia's correction which in combination to the absence of adverse cardiac events (e.g. torsades de pointes) suggests that it is not clinically significant (Bassat et al., 2009; Millat‐Martínez et al., 2018). In a large systematic review including data for 197,867 individuals who had received dihydroartemisinin plus piperaquine phosphate, there was only one reported case of potentially drug‐related sudden unexplained death, though no autopsy or ECG was done. The review concluded a median pooled risk estimate of sudden unexplained death after dihydroartemisinin plus piperaquine phosphate was 1 in 757,950, which was not significantly higher than baseline rate of sudden cardiac death (Chan et al., 2018). Similarly, another study of 2091 African patients enrolled in the WANECAM study found that dihydroartemisinin plus piperaquine phosphate, artesunate‐amodiaquine and artemether‐lumefantrine significantly prolonged QTc; however, there was no evidence of proarrhythmia (Funck‐Brentano et al., 2019). A randomised non‐inferiority trial in Kenya showed prolongation of QTc interval at 52 h was greater with arterolane‐piperaquine or arterolane‐piperaquine‐mefloquine than with artemether‐lumefantrine (Hamaluba et al., 2021). Interestingly, here the addition of mefloquine to arterolane‐piperaquine did not further increase the QTc interval (Hamaluba et al., 2021), which supports other clinical reports finding that mefloquine addition to dihydroartemisinin plus piperaquine phosphate did not further prolong QTc (van der Pluijm et al., 2020). However, adding amodiaquine to artemether‐lumefantrine did extend the QTc interval (van der Pluijm et al., 2020). The QTc interval was not significantly prolonged after treatment with artesunate alone but was significantly prolonged by combined artesunate with quinine treatment (Newton et al., 2001). Therefore, most clinical evidence indicated that artemisinin‐based combination therapy further prolong QT interval but that different artemisinin‐based combination therapy prolong the QT interval to different degrees. However, this was not associated with increased risk of cardiac arrhythmias such as torsades de pointes or sudden cardiac death.

Experimental studies investigating the molecular mechanisms for these interactions are exceedingly rare, however, it likely represents synergistic effects on ion channels. For example, the exaggerated QT interval prolonging effects of mefloquine‐halofantrine combination is the result of mefloquine's inhibition of I_Ks_ and halofantrine's inhibition of I_Kr_ thus inhibiting both delayed rectifier currents. This prevents the ability of the other current to compensate, reducing the ability of the myocardium to repolarise, resulting in significant QT prolongation (Geelen et al., 1999; Kang et al., 2001). Additive effects of combined therapy inhibiting the same channel, especially hERG, also likely play an important part in prolonging the QT interval (Penman et al., 2020). However, this has not yet been sufficiently demonstrated in the case of anti‐malarial drugs. For example, one study surprisingly reported that the combination of dihydroartemisinin plus piperaquine phosphate produced a level of hERG block, which was apparently less than that produced by piperaquine phosphate or dihydroartemisinin alone, but no firm explanation for this is available as of yet (Borsini et al., 2012).

### 4.3 Co‐morbidities and combination of anti‐malarial drugs with other drugs

Although treatment of uncomplicated malaria may seem relatively straightforward, this is not the case for the treatment of severe malaria or malaria in the presence of other co‐morbidities both of which necessitate additional treatments (Feachem et al., 2019; Flateau et al., 2011). This is important considering the majority of clinical studies investigating safety of anti‐malarial drugs, especially artemisinin‐based combination therapy, have been conducted in patients with uncomplicated malaria. Co‐morbidities may complicate treatment of malaria due to altered physiological responses to either the pathogen or drugs. These include pregnancy, HIV and diabetes mellitus (Flateau et al., 2011; Kwenti, 2018; Ngai et al., 2020). Table 4 outlines complications caused by these co‐morbidities.

**TABLE 4:** Complications caused by co‐morbidities in malaria

Co‐morbidity	Associated complication in malaria
HIV (Cohen et al., 2005; Flateau et al., 2011; Kwenti, 2018)	Increased risk of severe malaria
	Increased parasite load
	Increased risk of intensive care unit admission
	Anaemia
	Impaired treatment responses
	Increased reinfection risk
	Increased mortality
Pregnancy and HIV (Flateau et al., 2011; Kwenti, 2018; Villamor et al., 2005)	Increased parasitaemia
	Placental malaria
	Increased parasite densities (peripheral and placental)
	Anaemia
	Increased risk of maternal death
	Higher perinatal and postnatal mortality
	Low birthweight
	Preterm delivery
Pregnancy (Ngai et al., 2020; Thompson et al., 2020; Zakama & Gaw, 2019)	Preterm birth (gestational age <37 weeks)
	Foetal growth restriction; low birth weight and small for gestational age
	Risk of neurocognitive impairment
	Risk of later‐life neuropsychiatric disorders
	Increased risk of infant death; still birth
	Severe maternal anaemia and folic acid deficiency; leading to an increased risk of death from postpartum haemorrhage and heart failure
	Hypoglycaemia
	Kidney failure
	Pulmonary oedema
	Respiratory failure
	Secondary bacterial infections
	Increased risk of severe malaria
	Increased risk of maternal death

For example, patients infected with HIV are at a high risk of acquiring malaria with the risk being greater as immunity declines and in pregnant women (Flateau et al., 2011; Kwenti, 2018; Thompson et al., 2020; Villamor et al., 2005). Although some studies have shown that HIV‐infected patients have a higher risk of experiencing anti‐malarial treatment failure (Byakika‐Kibwika et al., 2010), the interaction between anti‐malarial drugs and anti‐retroviral drugs has not been well investigated and thus remains poorly understood. However, there is emerging evidence suggesting that anti‐retroviral drugs can influence anti‐malarial drug metabolism causing significantly increased anti‐malarial drug concentrations potentially exacerbating adverse proarrhythmic effects (Byakika‐Kibwika et al., 2010). For example, protease inhibitors like lopinavir/ritonavir are known potent inhibitors of cytochrome P450 (especially CYP3A4) metabolism. An open‐label study involving healthy HIV‐seronegative adults receiving combination treatment of artemether‐lumefantrine examined the effects of lopinavir/ritonavir on the pharmacokinetics of artemether‐lumefantrine. It found that lopinavir/ritonavir caused a 2‐ to 3‐fold increase in area under curve (AUC) for lumefantrine and altered artemether: dihydroartemisinin plus piperaquine phosphate (metabolite of artemether) AUC and C_max_ ratios (German et al., 2009). Contrastingly, other studies found that co‐treatment of artemether‐lumefantrine with efavirenz significantly decreased artemisinin and lumefantrine bioavailability and total exposure (E. Hughes et al., 2020), highlighting that different anti‐retroviral influence anti‐malarial drug metabolism differently.

Furthermore, there may also be possible synergistic effects on ion channel function. For example, atazanavir is a protease inhibitor and has been shown to prolong the QT interval via direct inhibition of the hERG channel by binding to its S6 domain as well as indirectly by obstructing hERG channel transport to the cell membrane (Cubeddu, 2016). Similarly, the protease inhibitors lopinavir and ritonavir have been observed to prolong the QT interval with reports of torsades de pointes in some patients which was associated with dose‐dependent inhibition of the hERG channel in HEK‐293 cells (Anson et al., 2005).

These findings suggest nuances in malaria treatment that may complicate assessment of arrhythmic potential of anti‐malarial drugs when treating the affected population, especially during efforts such as mass drug administration. Hence, the electrophysiological consequences of interactions between anti‐malarial drugs with other drugs used to treat common co‐morbidities such as HIV should be thoroughly investigated with both pre‐clinical laboratory and clinical studies.

## 5 CONCLUSIONS

Although some clinical studies have explored the cardiotoxic proarrhythmic potential of contemporary anti‐malarial drugs in isolation, larger phase IV studies are yet to be conducted pertaining to novel double and triple artemisinin‐based combination therapy. Mass drug administration is a key tool required to achieve the goal of malaria elimination, especially in the light of emerging multi‐drug resistant strains of parasite in endemic areas. Furthermore, there is significant paucity of data regarding the underlying proarrhythmic molecular mechanisms of anti‐malarial drugs. We clarify that anti‐malarial drugs can directly alter a variety of cell surface ionic currents (I_Na_, I_to_, I_Ca‐L_, I_Kr_, I_KATP_ and I_K1_) and disrupt intracellular Ca^2+^ handling and mitochondrial function causing increased ROS generation and tissue fibrosis. These changes are associated with the generation of early afterdepolarisation and delayed after depolarisation arrhythmic triggers, and the development of slowed conduction velocity and transmural dispersion of repolarisation arrhythmic substrates resulting in increased risk of arrhythmia. We also highlight areas where further potential proarrhythmic mechanisms are yet to be explored or where studies have revealed contradictory findings. Importantly, most of the proarrhythmic mechanisms described have been discussed in relation to quinidine and chloroquine due to absence of electrophysiological and molecular studies exploring newer anti‐malarial drugs individually or in combination. In addition to the potential synergistic effects of combinational therapy increasing arrhythmic risk, research should also explore how arrhythmic risk is altered in the presence of co‐morbidities commonly associated with malaria such as HIV.

### 5.1 Nomenclature of targets and ligands

Key protein targets and ligands in this article are hyperlinked to corresponding entries in the IUPHAR/BPS Guide to PHARMACOLOGY http://www.guidetopharmacology.org and are permanently archived in the Concise Guide to PHARMACOLOGY 2021/22 (Alexander, Fabbro, Kelly, Mathie, Peters, Veale, Armstrong, Faccenda, Harding, Pawson, Southan, Davies, Beuve et al., 2021, Alexander, Fabbro, Kelly, Mathie, Peters, Veale, Armstrong, Faccenda, Harding, Pawson, Southan, Davies, Boison et al., 2021, Alexander, Mathie et al., 2021).

## AUTHOR CONTRIBUTIONS

KS and NNK wrote first draft, reviewed and edited all subsequent drafts. ITF and CEE reviewed and edited all subsequent drafts. KJ reviewed all subsequent drafts, supervision and conceptualisation.

## CONFLICTS OF INTEREST

None declared.
